# Hematopoietic Stem Cell Gene Therapy for Cystinosis: From Bench-to-Bedside

**DOI:** 10.3390/cells10123273

**Published:** 2021-11-23

**Authors:** Stephanie Cherqui

**Affiliations:** Department of Pediatrics, Division of Genetics, University of California, La Jolla, San Diego, CA 92093, USA; scherqui@ucsd.edu; Tel.: +1-858-822-1023, Fax: 858-246-1125

**Keywords:** cystinosis, CD34^+^ hematopoietic stem and progenitor cells, gene therapy, pre-clinical studies, investigational new drug application, clinical trial

## Abstract

Cystinosis is an autosomal recessive metabolic disease that belongs to the family of lysosomal storage disorders. The gene involved is the *CTNS* gene that encodes cystinosin, a seven-transmembrane domain lysosomal protein, which is a proton-driven cystine transporter. Cystinosis is characterized by the lysosomal accumulation of cystine, a dimer of cysteine, in all the cells of the body leading to multi-organ failure, including the failure of the kidney, eye, thyroid, muscle, and pancreas, and eventually causing premature death in early adulthood. The current treatment is the drug cysteamine, which is onerous and expensive, and only delays the progression of the disease. Employing the mouse model of cystinosis, using Ctns^−/−^ mice, we first showed that the transplantation of syngeneic wild-type murine hematopoietic stem and progenitor cells (HSPCs) led to abundant tissue integration of bone marrow-derived cells, a significant decrease in tissue cystine accumulation, and long-term kidney, eye and thyroid preservation. To translate this result to a potential human therapeutic treatment, given the risks of mortality and morbidity associated with allogeneic HSPC transplantation, we developed an autologous transplantation approach of HSPCs modified ex vivo using a self-inactivated lentiviral vector to introduce a functional version of the *CTNS* cDNA, pCCL-CTNS, and showed its efficacy in Ctns^−/−^ mice. Based on these promising results, we held a pre-IND meeting with the Food and Drug Administration (FDA) to carry out the FDA agreed-upon pharmacological and toxicological studies for our therapeutic candidate, manufacturing development, production of the GMP lentiviral vector, design Phase 1/2 of the clinical trial, and filing of an IND application. Our IND was cleared by the FDA on 19 December 2018, to proceed to the clinical trial using CD34^+^ HSPCs from the G-CSF/plerixafor-mobilized peripheral blood stem cells of patients with cystinosis, modified by ex vivo transduction using the pCCL-CTNS vector (investigational product name: CTNS-RD-04). The clinical trial evaluated the safety and efficacy of CTNS-RD-04 and takes place at the University of California, San Diego (UCSD) and will include up to six patients affected with cystinosis. Following leukapheresis and cell manufacturing, the subjects undergo myeloablation before HSPC infusion. Patients also undergo comprehensive assessments before and after treatment to evaluate the impact of CTNS-RD-04 on the clinical outcomes and cystine and cystine crystal levels in the blood and tissues for 2 years. If successful, this treatment could be a one-time therapy that may eliminate or reduce renal deterioration as well as the long-term complications associated with cystinosis. In this review, we will describe the long path from bench-to-bedside for autologous HSPC gene therapy used to treat cystinosis.

## 1. Introduction

Cystinosis is an autosomal recessive disease that occurs in about 1 in 100,000–200,000 live births [1]. Cystinosis belongs to a family of lysosomal storage disorders and is characterized by the accumulation of cystine, the disulfide dimer of cysteine, within the lysosomes of all organs [2]. Three allelic forms of cystinosis exist. The most severe and the most common form of cystinosis is the infantile form of cystinosis (MIM #219800). The juvenile form (MIM #219900) is characterized by an adolescent onset of photophobia and glomerular renal impairment that can progress to end-stage renal failure. The ocular form (MIM #219750) is characterized by an adult onset of photophobia. Children affected with infantile cystinosis, thereafter referred to as cystinosis, appear normal at birth, but at 6–18 months of age, present for medical attention with a failure to thrive, polyuria, polydipsia, glucosuria, proteinuria, and often rickets. All of these symptoms are caused by renal tubular dysfunction (renal Fanconi syndrome) [3]. Cystinosis is currently the leading cause of inherited renal Fanconi syndrome in children, representing up to 20% of patients with hereditary tubular disorders [4]. Non-renal complications of cystinosis may manifest clinically in these patients overtime, including impairment of the heart, thyroid, muscles, pancreas, eyes, and central nervous system [5]. The main ocular manifestation is the crystal deposition in the cornea, which begins in infancy, increases with age, and gradually leads to pain, photophobia, blepharospasm, and recurrent corneal erosions [6]. Retinopathy can also be seen as early as 5 weeks of age [7], sometimes causing blindness [8,9]. Patients have increased risks of cardiovascular complications [10], diabetes mellitus, and hypothyroidism, and in males, hypogonadism with infertility [11]. Cystinosis also causes bone deformities and fragility [12], attributed to massive urinary phosphate loss, defective vitamin D conversion, and cystine deposition in the bones, although pathophysiology is not fully understood [13]. Cystinotic patients later develop neuromuscular and brain complications including fine vision deficits, poor motor coordination, peripheral muscle weakness, and swallowing dysfunction [14,15,16,17,18,19]. Distal myopathy can result into chronic respiratory dysfunction, swallowing difficulties, and aspiration pneumonia, a major cause of death [20,21]. Central nervous system (CNS) complications can also involve mental deterioration, impaired cognitive function, cerebral atrophy, seizures, and ischemic lesions [18,22,23].

Mutations or deletions in the ubiquitous gene *CTNS* cause cystinosis [24]. This gene encodes cystinosin, a seven-transmembrane domain lysosomal protein, which is a proton-driven cystine transporter [25,26]. Lysosomal cystine accumulation leads to the formation of cystine crystals, pathognomonic of cystinosis. In addition to lysosomal cystine storage, cellular dysfunctions, such as abnormal vesicular trafficking, autophagy, apoptosis, and TFEB (Transcription Factor EB) signaling, have also been described as responsible for the pathogenesis of cystinosis [27,28,29,30,31,32].

Despite early advancements in available therapies to delay the progression of cystinosis, including dialysis, renal transplantation, and cysteamine therapy, a significant unmet medical need still exists for patients. While dialysis and renal transplantation have enabled the survival of many children with cystinosis into adulthood, they are associated with significant challenges including life-long immunosuppressant intake. The cystine reduction therapy, cysteamine, is requires dosing every 6 h or every 12 h [33,34]. Although cysteamine has been shown to decrease white blood cell cystine levels, patients receiving long-term cysteamine therapy may continue to develop late complications such as hypothyroidism, diabetes, myopathy (including difficulty swallowing), and neurologic defects [35,36,37,38]. In addition, while cysteamine can delay the onset of complications such as end-stage renal failure, many of these patients ultimately progress and require a kidney transplant. The average age of mortality, even with long-term (>2 years) use of cysteamine, has been reported to be as young as 28.5 years [37], and the prognosis is largely dependent upon how early in the disease course cysteamine therapy is initiated [35,36,37]. Cysteamine is also associated with substantial untoward side effects including gastrointestinal pain and sulphurous body and breath odor; the odor leads to significant issues with compliance, especially in adolescents and young adults. In addition, patients must take multiple pills, up to 60 pills per day, around the clock.

As most of the organs are affected by cystinosis, functional cystinosin expression has to be reinstituted in the whole body. Hematopoietic stem and progenitor cells (HSPCs) gene therapy have emerged as an efficient therapeutic technology for genetic disorders of the blood system but also for parenchymal diseases as they are able to travel and engraft into injured tissues [39,40,41,42]. In the context of cystinosis, *CTNS* gene-modified hematopoietic stem and progenitor cells (HSPCs), upon autologous transplantation, are intended to engraft into the bone marrow, divide, and differentiate, thus providing a population of corrected cells that can supply functional cystinosin in the diseased organs for the life of the patient. Preclinical studies have been conducted in the mouse model of cystinosis, the Ctns^−/−^ mice [43,44]. The pre-clinical studies have shown the safety and efficacy of gene modified HSPCs for cystinosis leading to a clinical trial. If shown to be safe and effective for cystinosis, the one-time autologous transplantation of gene-modified HSPCs would represent a life-long therapy that has the potential to prevent kidney transplantation and long-term complications associated with cystinosis. Therefore, a Phase 1/2 study is currently being conducted at the University of California San Diego (UC San Diego) to evaluate the safety and efficacy of a single transplantation of autologous CD34^+^ enriched cell fractions transduced with a lentiviral vector containing the complementary deoxyribonucleic acid sequence that encodes for human cystinosin, the lysosomal cystine transporter protein (product name CTNS-RD-04), in patients with cystinosis. The path to go from bench-to-bedside is a colossal task that requires numerous people with regulatory, manufacturing, toxicology, and clinical expertise, as well as important funding support. Here, we review the path from the pre-clinical studies to the clinical trial for the hematopoietic stem cell gene therapy strategy for cystinosis.

## 2. Preclinical Proof of Concept and Mechanism of Action for Using HSPC for Cystinosis

As a preclinical proof of concept, we used the mouse model of cystinosis, using Ctns^−/−^ mice, a relevant model that recapitulates most of the main characteristics of the disease found in human cystinosis patients, such as mild renal Fanconi syndrome, chronic kidney disease, eye anomalies, and thyroid dysfunction [43,44,45,46,47]. As the source of HSPCs, we used the analogous murine stem cells antigen-1 (Sca1^+^) cells to the human CD34^+^ cells [48]. We showed that the transplantation of HSPCs expressing a functional *Ctns* gene isolated from congenic wild-type mice resulted in abundant tissue integration of bone marrow-derived cells, the significant decrease of cystine accumulation (up to 97% clearance), and long-term kidney preservation [49,50]. Indeed, while non-treated Ctns^−/−^ mice, or Ctns^−/−^ mice transplanted with Ctns^−/−^ mHSPCs, progressed to end-stage renal failure, age-matched Ctns^−/−^ mice transplanted with wild-type HSPCs maintained normal renal function and only focal histological kidney anomalies after more than a year post-transplant [50]. However, effective therapy depends on achieving a relatively high level of donor-derived *Ctns*-expressing cell engraftment (>50%). Finally, few to no cystine crystals were observed in the kidneys of treated mice, in contrast to non-treated Ctns^−/−^ mice, in which abundant cystine crystals were consistently observed in the kidney. We also demonstrated that HSPC transplantation was able to prevent eye defects in the Ctns^−/−^ mice [51]. Ctns^−/−^ mice with high engraftment levels (>50%) exhibited a dramatic reduction in crystal counts from the epithelial layer to the middle stroma (100% to 72% reduction, respectively), as well as normal corneal thickness and intraocular pressure as opposed to Ctns^−/−^ mice controls. This work was the first to demonstrate that transplanted HSPCs could rescue corneal defects and bring a new perspective to ocular regenerative medicine. The impact of transplanted HSPCs on the thyroid gland has been studied in collaboration with Dr. Pierre Courtoy (de Duve Institute, Belgium). Ctns^−/−^ mice present with sustained TSH activation combined with thyrocyte hypertrophy, hyperplasia, and vascular proliferation [52]. In contrast, Ctns^−/−^ mice treated with transplanted HSPCs exhibited normalization of cystine and TSH values and normal histology [53].

The extent of transplanted HSPC efficacy in cystinosis was surprising, especially considering that cystinosin is a transmembrane lysosomal protein as opposed to a secreted enzyme that can be recaptured by adjacent diseased cells [54,55,56]. In order to identify the cellular mechanism of action of this approach, we demonstrated for the first time that transplanted HSPCs, after differentiating into macrophages, transferred cystinosin-bearing lysosomes to the adjacent endogenous host cells via tunneling nanotubes (TNTs) [57]. We also demonstrated in vitro that Ctns-deficient cells exploited the same route to retrogradely transfer cystine-loaded lysosomes to macrophages, providing a bidirectional correction mechanism. This bidirectional exchange, allowing the clearance of the lysosomal cystine load in both cell types, probably accounts for the robust decrease in cystine levels observed in all tissues in the HSPC-transplanted Ctns^−/−^ mice [49]. TNT formation was enhanced by the presence of diseased cells [57,58]. In vivo macrophage-derived tubular extensions penetrated the dense tubular basement membrane and directly delivered cystinosin-containing vesicles into the epithelia in Ctns^−/−^ mice, preventing proximal tubular cell degeneration [57]. Macrophages were also observed in the cornea (and retina) as the primary differentiated cells from the transplanted HSPCs and transferred cystinosin-bearing lysosomes via TNTs to keratocytes [51]. TNTs were also observed in the transplanted HSPC-derived cells engrafted in the thyroid [53]. This was the first proof of concept of a genetic lysosomal defect correction by bidirectional vesicular exchange via TNTs, suggesting broader potential for HSPC transplantation for the treatment of other disorders due to defective vesicular proteins.

## 3. Ex Vivo Gene Modified Cell Therapy: A Safer Approach Than Allogeneic Transplantation

Given the considerable risk of morbidity and mortality associated with allogeneic HSPC transplantation, it remains an uncertain therapeutic choice for many diseases after the consideration of the risk/benefit ratio. The major complication is graft-versus-host disease (GVHD) [59,60]. Acute GVHD grade II-IV occurred in 20% to 32% of patients and chronic GVHD in 16% to 59%, both significantly impacting the survival of the recipients [61,62,63]. Allogeneic hematopoietic stem cell transplantation (HSCT) has been performed on one patient affected with cystinosis [64]. The patient was a 16-year-old Caucasian male, who was diagnosed with cystinosis at the age of 2.7 years and immediately treated with cysteamine. The patient underwent allogeneic HSCT from a full HLA-matched unrelated donor. Cysteamine treatment was discontinued 2 months prior to transplantation. For post-transplant immunosuppression, tacrolimus, mycophenolate mofetil, methotrexate, and prednisolone were used. Acute GVHD and adenovirus reactivation developed during the third week following HSPC transplantation, presenting with fever and profound diarrhea. Due to partial graft failure, a second HSPC infusion from the same donor was administered 15 months after the first HSCT, resulting in a higher yield of engraftment. However, a severe therapy-resistant chronic cutaneous, gastro-intestinal, and liver GVHD developed, for which several immunosuppressive agents were applied, including prednisolone, azathioprine, cyclosporine, ATG, and sirolimus. The patient died 35 months after transplantation from severe pneumonia due to a multi-resistant Pseudomonas infection. Despite GVHD and other severe adverse events due to the allogenic transplant, efficacy of HSPC transplantation on cystinosis was demonstrated. During the first few months post-HSPC transplant, kidney function stabilized, and polyuria decreased. Patient’s photophobia score improved from grade 5 to no photophobia. Stomach biopsies, taken after transplantation, showed a significant decrease in cystine crystal accumulation. The cDNA derived from patient’s tubular epithelial cells collected from urine and liver biopsy taken at 24 months post-HSC transplantation showed donor HSPC-derived tissue engraftment of 22% and 40%, respectively. This dramatic case report underlines that HSPC transplantation holds the potential for therapeutic benefits for cystinosis with the restoration of *CTNS* expression in tissues, a decrease in cystine crystal accumulation, and the improvement of polyuria and photophobia. However, this case also strongly highlights the high risks associated allogeneic HSC transplantation with a potentially lethal outcome.

In contrast, autologous HSPC gene therapy morbidity is substantially lower because it abrogates the risk of GVHD and immune rejection. Another key advantage of this approach is that no immunosuppressants are necessary after transplantation. For HSPC gene therapy, the patients’ own HSPCs are ex vivo gene-modified to correct the gene defect. Patients still require myeloablation conditioning but at a reduced intensity. Inherent risks in this approach reside in the use of retroviral vectors to bring the normal copy of the gene and these vectors will integrate within the genome. Cases of leukemogenic complications were reported in clinical trials for severe combined immunodeficiency-X1 (SCID-X1) using autologous HSPCs and the murine leukemia virus (MLV) vector [65]. Extensive investigation of this issue revealed that MLV vector was preferentially integrated near cancer-implicated genes such as CCND2 and HMGA2, and the long terminal repeats (LTR) of the vectors contained strong enhancer/promoters that triggered the distant enhancer activation of these genes [66]. Since then, MLV vectors have been supplanted by a lentiviral vector (LV) for ex vivo gene modification of HSPCs because of their safety. The third generation of LV, self-inactivated (SIN)-lentiviral vectors, have engineered LTRs which removes their very strong promoter/enhancer activity. Therefore, an internal promoter is added in order to drive the therapeutic gene (transgene). These promoters are far less potent which reduces the potential risk of interactions with nearby cellular genes and thus, enhances safety [67,68]. Moreover, in contrast to the MLV, lentiviral vectors are not associated with oncogenesis and therefore may represent a safety advantage over oncoretroviral gene therapy vectors. In the last 12 years, 400–500 patients have been treated with lentiviral ex vivo gene therapy, including three regulatory approved products in Europe for thalassemia, metachromatic leukodystrophy, and cerebral-adrenoleukodystrophy (C-ALD) [56,69,70,71,72,73,74]. Thousands more oncology patients have been treated with lentiviral-transduced T cells, known as CARTs. All these patients are closely followed up. There have been no reported cases of oncogenesis until two recent cases. Both were in a Bluebird Bio C-ALD clinical trial. The two patients developed dominant clonal expansions within a year of gene therapy infusion, with persistent thrombocytopenia and dysplastic (abnormal) megakaryocytes. A diagnosis of myelodysplastic syndrome of single lineage dysplasia (MDS-SLD) was made. This type of MDS is not common, and seldom, if ever, progresses to acute myeloid leukemia. Patients often live a long time even without treatment. The root cause of the MDS-SLDs is at present unknown and under investigation. As a result of the many differences between all the lentiviral ex vivo gene therapies, the two cases of MDS-SLD are considered specific to this clinical trial. Overall, lentiviral vectors continue to demonstrate a significant safety advantage over gamma-retroviral vectors.

## 4. Preclinical Studies for the Autologous Gene-Modified Hematopoietic Stem Cell Approach for Cystinosis

The lentiviral vector backbone we used for cystinosis is pCCL-EFS-X-WPRE [75], which was provided by Dr. Donald Kohn who used the same vector for the (ADA)-Deficient Severe Combined Immunodeficiency (SCID) clinical trial [76]. This is a third generation self-inactivating (SIN)-lentiviral vector. A central polypurine tract (cPPT) fragment that increases the nuclear import of viral DNA was added to the CCL vector backbone [77]. A woodchuck hepatitis virus posttranslational regulatory element (WPRE) is present to boost titer and gene expression. However, its open-reading frame was eliminated [78], as it overlapped with the woodchuck hepatitis virus X protein, a transcriptional activator involved in the development of liver tumors [79]. The transgene expression is driven by the ubiquitously expressed short intron-less human Elongation Factor 1 alpha promoter (EFS, 242 bp) [80]. The EFS promoter, which lacks the intron and enhancers of the larger elements used in many expression plasmids, has been shown to direct high level transcription of reporter genes in murine HSCs and to have significantly reduced *trans*-activation potential compared to γ-retroviral LTR [81]. We subcloned the human *CTNS* complementary deoxyribonucleic acid (cDNA) from the starting codon (ATG) to the stop codon (TAG) in the pCCL-EFS-X-WPRE lentiviral vector (pCCL-CTNS).

We performed the first proof-of-concept in the mouse model of cystinosis, the *Ctns^−/−^* mice, believing that autologous gene-modified HSC transplantation using a SIN-LV could work. We worked with the human *CTNS* gene and the vector backbone (pCCL-CTNS) to create the preclinical proof-of-concept for a human trial. The analogous cells of the human CD34^+^ HSPCs are the murine Sca1^+^ HSPCs. We used freshly isolated Sca1^+^ HSPCs from *Ctns^−/−^* mice [82]. We optimized an efficient transduction protocol for the murine HSPCs that could achieve >80% of transduced cells and showed that pCCL-CTNS-transduced HSPCs kept their capacity to engraft efficiently into all organs with a long-term expression of the transgene [82]. Then, we showed that transduced cells were capable of decreasing cystine content in all tissues and improving kidney function in the Ctns^−/−^ mice [82].

## 5. Investigational New Drug-Enabling Studies

**Figure 1:**
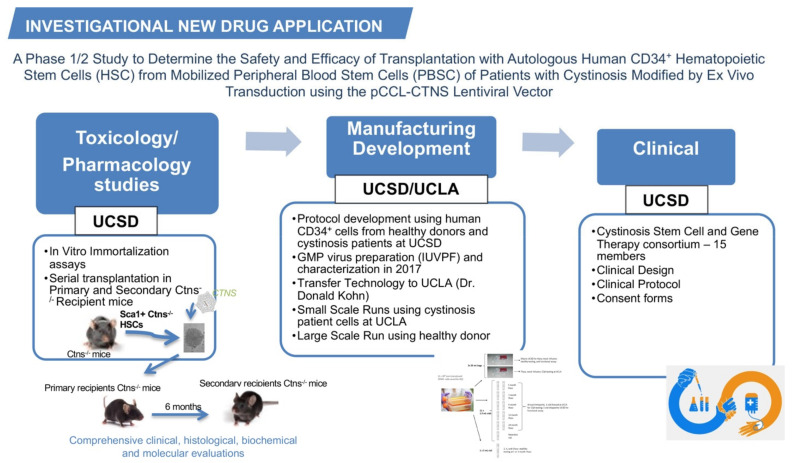
The investigational new drug application for cystinosis. The investigational new drug application contains three main categories, the toxicology/pharmacology studies, the manufacturing development, and the clinical design. The main studies performed in each category are listed in the figure.

Based on our preclinical results, we submitted a pre-investigational new drug (IND) application regarding the gene-modified HSPCs for cystinosis in March 2013 and had the first teleconference with the Food and Drug Administration (FDA) one month later. We proposed a plan for the pharmacology/toxicology, manufacturing development, and clinical design for the future clinical trial; these three categories composing the IND application (Figure 1). Based on FDA feedback, we designed the studies necessary for inclusion in an IND.

### 5.1. Pharmacology/Toxicology Studies

CTNS-RD-04 consists of CD34^+^ enriched HSPCs that underwent ex vivo transduction using the pCCL-CTNS that carries the full-sequence, human cystinosis gene (*CTNS*) cDNA. As the use of human HSPCs for nonclinical experimentation is not feasible in the Ctns^−/−^ mouse disease model, due to xenogeneic reaction towards human stem cells, the in vivo characterization of pCCL-CTNS construct functionality was performed and the murine analogous cells Sca1^+^ HSPCs were isolated from the Ctns^−/−^ mice. Studies were designed as a serial transplantation study, with either pCCL-CTNS-transduced (test article) or mock-transduced (control article) murine Ctns^−/−^ Sca1^+^ HSPCs transplanted into primary recipient Ctns^−/−^ mice; then, at 6 months post-transplant, bone marrow from the primary recipients was transplanted into secondary recipient Ctns^−/−^ mice. We conducted the pharmacology/toxicology studies for the autologous transplantation of HSPCs ex vivo gene-modified with pCCL-CTNS using a batch of pCCL-CTNS lentiviral vector preparation produced under comparable good manufacturing practice (GMPc). Toxicity was determined by comprehensive clinical and histological tissue analyses and the determination of vector copy number (VCN) in blood, bone marrow, and hematopoietic lineage cells; VCN representing the average of vector copies per cell. Treated mice exhibited normal kidney function, and no indications of toxicity or histological anomalies attributable to the transplanted pCCL-CTNS-transduced HSPCs. Sustained multilineage hematopoietic cell transduction was obtained, and *CTNS* expression was detected in all tissues tested in primary test male and female mice. Finally, tissue cystine levels were reduced in most of the tissues in the primary test male and female mice compared to control male and female mice, demonstrating test article efficacy. Persistence of gene marking and *CTNS* expression in the secondary transplanted mice showed the capacity of the pCCL-CTNS vector in transducing primitive repopulating murine HSPCs. In addition, a vector integration site (VIS) analysis was performed by Dr. Frederic Bushman at the University of Pennsylvania and did not detect enrichment of integration events near oncogenes; the frequency of integration sites near oncogenes in bone marrow cells in the recipient mice was generally less than that of mice in a previously published thalassemia mouse study from which no adverse events have been reported [83]. Lastly, the potential genotoxicity associated with the pCCL-CTNS lentiviral vector was analyzed by two in vitro immortalization assay (IVIM) analyses performed in two independent laboratories, which detected transformed clones of murine bone marrow cells by insertional mutagenesis arising after multiple plating of the cells [84]. These assays demonstrated that the lentiviral vector, pCCL-CTNS, did not show growth of insertional mutants, suggesting the absence of vector associated genotoxicity, even at high vector copies per cell (up to VCN 16.9), and does not possess significant transforming capability in this in vitro assay. Altogether, the nonclinical data (pharmacology, distribution, and toxicology) collected in the Ctns^−/−^ mice, indicate that CTNS-RD-04 was expected to be safe when administered in human subjects and has the potential to improve patients’ welfare.

### 5.2. Manufacturing Development

We optimized a protocol to transduce human CD34^+^ hematopoietic stem cells with our lentiviral vector to obtain a VCN included between one and five. We performed colony forming unit (CFU) assays using human CD34^+^ peripheral blood stem cells (PBSC) isolated from five healthy donors and four cystinotic patients. No aberrant proliferation or differentiation potential with the lentivirus compared to negative controls was observed in any of these assays. Moreover, vector integration site (VIS) analysis in the patient’s cells showed no enrichment of the integration sites near proto-oncogene 5′ ends. The clinical grade pCCL-CTNS virus preparation was produced as a ~60 L full-scale preparation at the Gene Therapy Resources Program (GTRP), Clinical Grade Lentivirus Vector Core (Indiana University National Gene Vector Laboratory) directed by Dr. Kenneth Cornetta who also prepared the good manufacturing practice (GMP)-comparable virus used for the toxicology studies. Technology transfer, small-scale runs, and large-scale runs using the GMP pCCL-CTNS lentiviral vector preparation were then performed at the GMP Human Gene and Cell Therapy Facility (HGCTF) at the University of California, Los Angeles (UCLA) directed by Dr. Donald Kohn where the manufacturing of the patients’ cell product is currently taking place. Stability of the GMP pCCL-CTNS virus vector and investigational product have been established.

### 5.3. Clinical Design

The clinical protocol was designed by the Cystinosis Stem Cell Gene Therapy Consortium composed of 15 members including 13 clinicians in the field of bone marrow transplant, nephrology, metabolic, gastroenterology, neurology, ophthalmology, and dermatology.

The toxicology/pharmacology studies, manufacturing development, and clinical design have been reported in an IND application that was submitted to FDA on 19 November 2018, and we received clearance to proceed to a phase 1/2 clinical trial in 19 December 2018. A Data Safety Monitoring Board was established to review the protocol and provide subsequent trial oversight.

## 6. Autologous Gene-Modified HSPC Clinical Trial for Cystinosis

**Figure 2:**
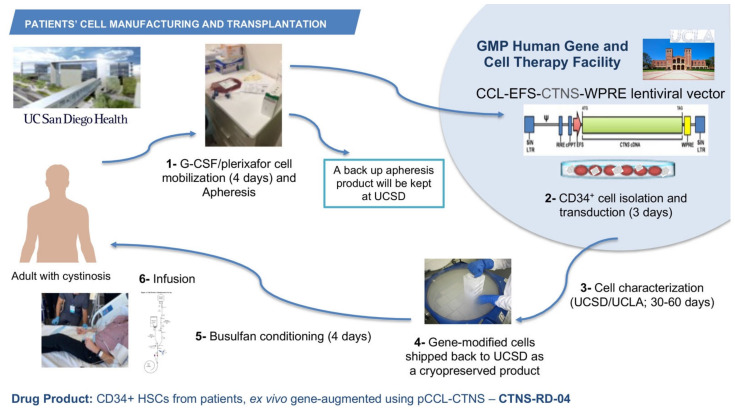
Manufacturing and infusion of the investigational product CTNS-RD-004. Patients with cystinosis undergo G-CSF/plerixafor stem cell mobilization in the peripheral blood, and then apheresis. One bag of apheresis is kept at UC San Diego and one bag is shipped to the HGCTF at UCLA, where the CD34^+^ cells are isolated and transduced with the GMP pCCL-CTNS virus preparation. The cell product is then cryopreserved and characterized. If the product meets the release criteria, it is shipped to UC San Diego where it is infused to the patient.

The phase 1/2 open-label, first-in-human clinical trial started in July 2019 after receiving funding from the California Institute of Regenerative Medicine (CIRM), the Cystinosis Research Foundation, and the National Institute of Health (NIH). The investigational product CTNS-RD-04 consists of the gene-modified CD34^+^ enriched HSPCs. A target of six patients will be enrolled staggered by cohort of two patients; the two first cohorts include adult male, or females and the third cohort may include adolescents. The primary objective is to assess the clinical tolerability and safety of the treatment of CTNS-RD-04. The secondary objective is to evaluate the impact of treatment with CTNS-RD-04 on cystine levels and cystine crystal counts in the intestinal mucosa and skin, cystine crystal counts in the cornea and cystine level on white blood cells and to evaluate the effect of treatment on clinical disease outcomes, including kidney function, vision, muscle strength, respiratory function, bone density, muscle mass, endocrine function, and quality of life. As a non-invasive way to image and quantify cystine crystals in the skin, we developed and optimized a new method using intradermal confocal microscopy and an advanced imaging software [85]. The inclusion criteria include a diagnosis of infantile cystinosis, a glomerular filtration rate (GFR) > 15 mL/min/1.73 m^2^, adequate thyroid function (TSH: 0.27–4.2 mIU/L, and T4 < 2 × ULN mcg/dL), and adequate respiratory function (FEV1 > 50%). For patients who underwent a kidney transplant, one-year post-transplant is required for enrolling. After provision of informed consent, subjects undergo screening and baseline clinical, histological and biochemical evaluations to determine health status, study eligibility, and blood and tissue cystine levels. The use of oral cysteamine is interrupted 2 weeks before baseline assessments and subsequently resumed. Eligible subjects undergo granulocyte colony stimulating factor (G-CSF) and plerixafor-mediated peripheral blood stem cell (PBSC) mobilization, and apheresis. Plerixafor is a small-molecule antagonist of CXCR4 and CXCL12-mediated chemotaxis, and the combination G-CSF/plerixafor has been shown to improve mobilization and the cells display a more primitive stem cell phenotype [86]. One apheresis bag is kept at UC San Diego as backup, and one bag is transported via a certified carrier to HGCTF at UCLA (Figure 2). CD34^+^ cells are isolated, transduced using the pCCL-CTNS lentiviral vector, and cryopreserved while the cellular investigational product is being characterized (Figure 2). Once the investigational product meets release criteria, subjects discontinue oral cysteamine therapy 2 weeks prior to reduced intensity myeloablation conditioning because the impact of cysteamine on conditioning and bone marrow reconstitution is unknown. The dosing for myeloablation of the clinical trial is completed using busulfan, which is not nephrotoxic so is particularly important in the case of patients with cystinosis. CTNS-RD-04 is infused once intravenously, at the minimal dose of 3 × 10^6^ CD34^+^ cells/kg (Figure 2). Subjects discontinue cysteamine eye drops 1-month post-infusion. Follow-up visits for clinical, histological, and biochemical evaluations occur at approximately 3-, 6-, 12-, 18-, and 24-month post-transplant. CTNS-RD-04 will engraft into the bone marrow, divide, and differentiate, thus providing a population of corrected circulating cells with normal *CTNS* encoding cystinosin. Corrected cells are expected to engraft in cystinosis-mediated injured tissues, and the production of the normal protein is expected to cross-correct other cells in the body via tunnelling nanotubes. By allowing restoration of functional cystinosin, it is anticipated that cell survival will improve and ultimately lead to reduction in morbidity and early mortality in cystinosis patients. Three patients affected with cystinosis have been infused so far, the first patient was transplanted on 7 October 2019. Assessment of the outcomes are currently being evaluated. A partnership has been established with the ex vivo gene therapy biotech company, AVROBIO, Inc, that is currently planning to conduct a phase 3 clinical trial.

## 7. Conclusions

Significant medical unmet needs still exist for patients with cystinosis. Autologous hematopoietic stem cell gene therapy represents a promising new therapeutic approach for the treatment of cystinosis that may have the potential to address most of the complications associated with this disease, and the current clinical trial will assess its potential in patients. The path from bench-to-bedside took a village, high financial support, and many years. Alongside this, the presence of the Cystinosis Research Foundation advocacy group has been instrumental in the successful clinical translation of this project.
